# Investigating the relationship between free chlorine concentration and heterotrophs in water of swimming pool in Iran

**DOI:** 10.1016/j.heliyon.2024.e36804

**Published:** 2024-08-23

**Authors:** Roshanak Rezaei Kalantary, Yasaman Oshidari, Vida Amoohadi, Hadi Niknejad, Reza Zeraatkar, Mohsen Hesami Arani

**Affiliations:** a Department of Environmental Health, School of Public Health, Iran University of Medical Sciences, Tehran, Iran; b Student Research Committee, Department of Environmental Engineering, School of Public Health and Safety, Shahid Beheshti University of Medical Sciences, Tehran, Iran; c Health System Research Council, Deputy of Health, Kashan University of Medical Sciences, Kashan, Iran; d Social Determinants of Health (SDH) Research Center, Kashan University of Medical Sciences, Kashan, Iran; e Research Center for Environmental Health Technology, Iran University of Medical Sciences, Tehran, Iran

**Keywords:** Free Chlorine, Iran, Swimming pool, Water

## Abstract

Swimming pools rank high among dangerous places for human recreation, which play an important role due to the high number of references and require continuous monitoring of pollutants and factors affecting it. Therefore, this study was conducted with the aim of systematically investigating the relationship between chlorine and heterotrophs in published studies in Iran. PubMed, Science Direct, and Google Scholar databases were searched from April 2000 to September 2022. Then identified studies were evaluated, then the studies lacking the necessary quality were excluded. From 686 articles, 33 reputable studied investigated the concentration of free chlorine in Iranian pool water. However, 14 studies measured the free chlorine and heterotrophs in swimming pools of 12 different Iranian cities. According to the results of the present study, the appropriate residual chlorine has reduced the microbial contamination of the pools, also, using the clarification in conjunction with the remaining free chlorine concentration, can help ensure the bacteriological quality of the water in the swimming pool. So maintaining the residual chlorine levels and daily cleaning of the pool can be effective in controlling heterotrophs in swimming pools and water recreational environments.

## 1 Introduction

Swimming pools are one of the most popular recreational and sports centers that can be a potential source of contamination due to the presence of different segments of society [1,2]. The amount of this contamination is due to lack of maintenance, monitoring and inadequate treatment, increased use of swimming pools, and adding materials from swimmers to water such as hair, fatty oils, excretion, respiratory, gastrointestinal, genital, and other harmful bacteria. It increases rapidly and as a result, long term contact with water causes the transmission and prevalence of fungal, parasitic and bacterial diseases [[3], [4], [5]].

Several diseases related to swimming pools was reported in many studies that included: gastrointestinal diseases, typhoid, diarrhea, and cholera, eye and nose diseases, purulent sore throat, ear pain, conjunctivitis, skin diseases, yellow wounds and so on [[6], [7], [8]]. Therefore, it is necessary to bring attention to these health outcomes as a result of exposure to the swimming pool environment to ensure that the water in these facilities are being treated appropriately to ensure the public health and safety of the swimmers and users of these swimming pools. One of the most important hygiene issues is to pay attention to the quality of water consumed so that the water supply must have appropriate physical, chemical, and microbial properties and maintain the desired level during the use of water. It is also important for the consumer to maintain the appearance and aesthetic aspects [9]. Swimming pool source waters are usually supplied from ground water wells or from finished drinking water sources which are usually treated with various disinfectants such as chloride, bromide, iodine, ozone, and UV radiation lamps [5,6,10]. Chlorine and its derivatives are the most common disinfectants for water use compared to other disinfectants due to their low cost, effectiveness, and easy application [11,12]. Bromide and iodine are not frequently used. Chlorine and its derivate are the most commonly used as it leaves a residual. Disinfection by ozone or UV does not leave a residual and is very difficult to determine what is left in the final water sample. The price of granular sodium hypochlorite 10 % has greatly increased in price since the late 1980s. Due to supply chain issues and consolidation of manufacturers, this product is now costly in some parts of the world.

Therefore, in order to ensure public health and observance of standards, the pool water needs to be tested to determine the remaining free chlorine. In fact, the proper and regular chlorination of swimming pools along with health supervision by health officials and regular chlorine measuring with pH values can be a useful tool to ensure that the aforementioned diseases are not infected [12]. The most important physical and chemical factors involved in the spread of skin diseases in the pools are the pH, the remaining chlorine, the turbidity, and temperature which play a significant role in describing the microbial quality of the pools [13]. The standard range for the pH of the pool water between 7.2 and 7.8 and the remaining chlorine residue is 1–3 mg/L, Water temperature 25-29 °C and opacity is maximum 0.5 NTU. Failure to comply with each of these cases increases the activity of microorganisms in watera [14,15]. The most important microorganisms in pool water are *fecal bacteria*, *Pseudomonas aeruginosa, fecal Streptococcus*, and *mycobacterium marinum*, known as swimming pool water pollutants, although according to the World Health Organization, microbial to evaluate microbial conditions used are heterotroph bacteria [1,13]. Heterotroph bacteria are a set of optional aerobic and anaerobic bacteria that are not capable of synthesizing all the nutrients they need. Therefore, they are dependent on the external source of organic and non-organic matter for nutrition [16]. These bacteria include *aerobic* and *anaerobic bacteria*. In this group, there are non-pathogenic and sometimes opportunistic bacteria (*Pseudomonas bacteria*) and pathogenic (*Escherichia coli and Aeromonas*) that often gram negative, cause diseases such as urinary tract infection, respiratory system, gastric and intestinal infections, and various systemic infections [17]. These bacteria are found in sand and ripple films, water distribution networks and their components, such as cooling tower, pressure tanks, and wall bottles [18,19]. Determining the presence of *Coliform* and *Streptococcus bacteria* in water and adapting to existing standards is a common indicator of microbial quality of drinking and recreational water [20]. Although the coliforms are stool pollution indicators and *Staphylococcus aureus* and *Pseudomonas aeruginosa* are considered an indicator of pool water risks, *heterotrophic bacteria (HPC)* was considered the most important indicator of water disinfection efficiency for water quality control [9]. In this regard, various studies have examined the presence of fecal coliforms and heterotrophs in the pool water. In a study conducted by Firouzi et al., 2020, compared the microbial quality of disinfectant swimming pools with the process of ozone and chlorine in Tabriz [10]. In another study conducted by Fadai et al., In 2013, aimed at comparing the chemical, biological and physical quality of swimming pools (18). Also, a study by Tehran University of Medical Sciences was conducted on the water of Tehran University of Medical Sciences, the results showed that in 48 % of the total samples, the free chlorine remained and in 76.1 % of the samples, the pH was in the standard range and also in 78.8 % of *heterotrophic bacteria* was desirable [21]. Due to the sensitivity of the subject, this systematic review study was investigated the relationship between chlorine concentration and heterotrophs in water of swimming pool in studies published in Iran.

## 2 Method

The present study is a systematic review study that examines the amount of chlorine-free concentration and heterotrophs in the water of swimming pools in Iran in existing studies.

The main question was: what is the relationship between the concentration of chlorine and heterotrophs in the water of swimming pools in Iran? Articles that measured the amount of free chlorine in the pool and heterotrophs in Iranian recreational pools were examined. Therefore, a search strategy was designed to identify articles related to the main topic.

### 2.1 Search strategy

**Fig. 1:**
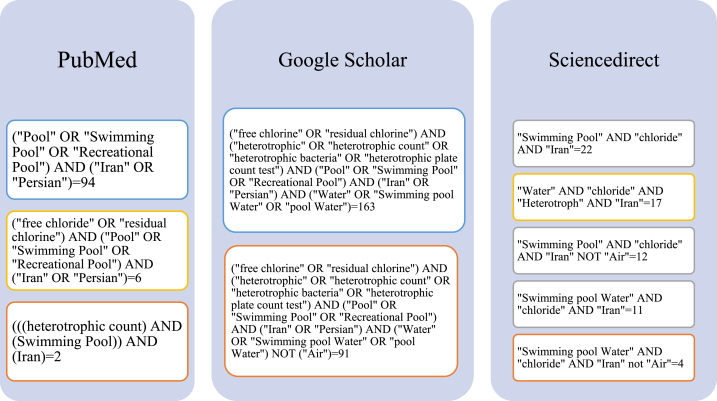
Different forms of search strategies in in PubMed, Science direct, and Google Scholar databases.

In the first step, the search for articles was performed in a systematic review in PubMed, Science direct, and Google Scholar databases based on keywords. The search strategy was a combination of the following key words which was placed in databases in different forms: ("free chloride" OR "residual chlorine") AND ("*heterotroph*" OR "heterotrophic count" OR "*heterotrophic bacteria*" OR "heterotrophic plate count test") AND ("Pool" OR "Swimming Pool" OR "Recreational Pool") AND ("Iran" OR "Persian") AND ("Water" OR "Swimming pool Water" OR "pool Water") NOT ("Air") (Fig. 1)**.**

### 2.2 Screening

Initially, the keywords of search strategy was searched in the database and in the extracted relevant papers by investigating the titles and abstracts. Then, two reviewers critically and independently reviewed and evaluated all identified papers (the titles, abstract, and full text) for eligibility.

### 2.3 Selection criteria

As inclusion criteria: the selected papers for this review were included: a) papers that have simultaneously measured the residual free chlorine in the pool water and counted the heterotrophs, b) papers that have published in reputable journals.

Exclusion criteria: a) the papers that did not have clear results, b) papers that had performed measurements in the air or drinking water of the swimming pool, and c) papers that had taken samples from natural swimming pools; were excluded from this study.

### 2.4 Quality assessment

The quality assessment of the studies was done using the quality assessment checklist. At this stage, among the available articles, 2 papers were excluded from the study due to not meeting the minimum quality standards.

### 2.5 Study selection

**Fig. 2:**
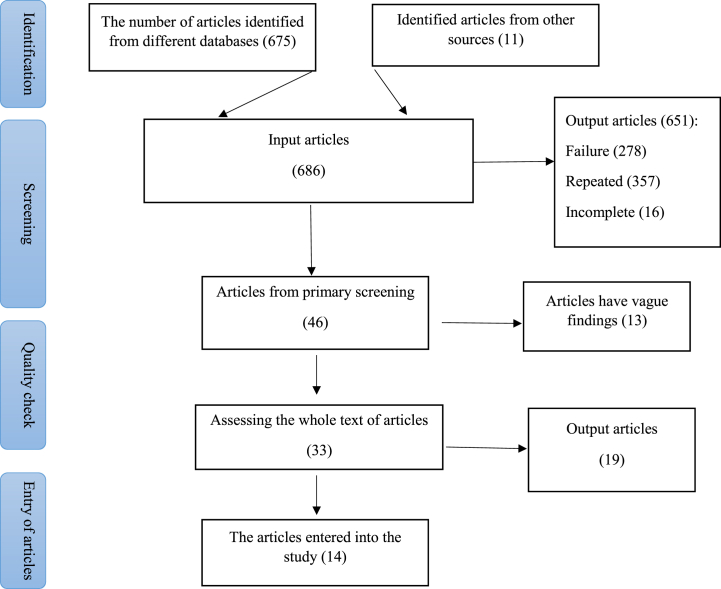
Flowchart of search in information resources, selection and review of articles.

The papers was screened independently by authors, based on the criteria mentioned above. After the initial screening of the titles, 686 studies were selected (Fig. 2). In the case of conflicting decisions over the initial screening, the respective study was included in the next step of screening. Finally, the contents of articles were studied, and 33 papers have been selected for this reason, which contained sufficient information relevant to the purpose of the study (Fig. 2).

## 3 Result

In this systematic review study, the published papers in the years 2000–2022 were reviewed. Out of 686 articles, 46 papers were studied the concentration of free chlorine in Iranian pool water in different seasons. 14 studies of these 46 articles were measured free chlorine and heterotrophs in 120 swimming pools of 12 different cities (Table 1). And also 19 of these 46 articles were done in 230 Iranian pools. In these articles only chlorine concentration in pool water were reported (Table 2). A lot of repeated studies also lacked necessary information, and some studies had incomplete quality and were excluded from this systematic review study (Fig. 2). Out of 33 entry studies, 15 studies were done in central cities of Iran, 8 studies in the western cities, 5 studies of the Northern provinces, 1 study related to the eastern provinces, and 3 studies were related to the southern provinces. 30 % of studies were conducted in 4 seasons, 2 studies in 3 seasons, and the other 2 seasons or less. According to the purpose of the study, 14 studies in Table 1 were eligible for systematic study, out of which 14 of these studies have expressed 3 correlation (r) studies between the residual free chlorine and the amount of heterotrophy, which this number is between 0.14 and 0.59. More than 2203 pool water samples were studied in these 14 studies, and the mean number of heterotrophic in 12 input studies (up to 200 ml) and 2 studies was higher than the standard, the final average heterotrophy number The ones was 95.77 ± 31 in these 14 studies.

**Table 1:** Simultaneous measurement studies of free chlorine and heterotrophs in swimming pool water.

City name	Pools Qty	sample number	free chlorine average (mg/L)	turbidity NTU Mean ± st dev	Heterotroph HPC (CFU)	pH average	season	author	Classification Year	Publish year
Esfahan	25	234	1.2 ± 0.7	0.2 ± 0.4	68	6.1–9.4	4 seasons	Mahnaz nick aeen	2006	2010
Shahin shahr	3	288	0.77 ± 0.76	0.5 ± 0.2	17.376	<8	Winter and spring	Somayeh rezaii	2011	2015
Karaj	7	91	2.2	…	190	7.5	25 may until august	Munes Asady	2013	2015
Tehran	All pools		1.8	0.82 ± 18	154.5	6.8–8.4	1 year	Ayub beyki	2013	2016
Khorram abad	All pools	60	0.957 ± 2.65	0.85 ± 0.9	0.48 ± 71	0.43 ± 0.267	3 months	Bahram kamrei	2015	2018
karaj	35	315	0.79 ± 1.891	0.9 ± 0.3	11.18	6 ± 0.284 7.1	9 months	Akbar eslami	2015	2016
kerman	10	150	0.5–1.2	0.1–0.5	0.5–1.2	6.5–8.5	Summer, autumn and winter	Hossein Jafari mansourian	2011	2013
Urmia	14	336	0.846 ± 1.6	0.4 ± 02	0.846 ± 1.6	57 ± 0.317	Summer and autumn	Mohammad ali zazouli	2013	2014
Yazd	7	42	0.628	0.34 ± 0.37	0.628	8 ± 0.246 7.8	summer	Mohammad taghi ghaneian	2011	2013
damghan	2	36	1.97	1.7 ± 0.7	1.97	7.6	1 year	Khalil allah moeinian	2012	2015
Sanandaj	9	108	0.39 ± 1.5	0.48 ± 0.093	0.39 ± 1.5	0.5 ± 0.277	Summer	Pegah Bahmani	2013	2014
Shahrak rad	2	459	0.63 ± 0.15 and 0.58 ± 0.19	1 ± 0.15 and 1.85 ± 0.25	1.2 ± 0.55 and 2.2 ± 0.06	8.08 ± 0.29 and 7.85 ± 0.24	Summer and winter	A fadaei	2013	2014
Tabriz	2	24	2.2	0.45	200–480	7.8	Summer, autumn and winter	Parisa firozi		2020
Kerman shah	5	60	0.92	1 ± 0.3	75.4	6.88	ND	Amir karami		2015

**Table 2:** Studies on measuring free chlorine in swimming pool water without reporting heterotrophs.

City	Pools Qty	sample number	free chlorine average (mg/L)	pH average	season	author	Classification Year	Publish year
Mashhad	26	312	0.461		4 seasons	Azam jabery	1998–2003	2009
Tehran	27	113	1.15 ± 1.26	7.69 ± 0.37	Spring and summer	Mohammad hadi dehghani	2013	2015
Kerman	10	1500	1.6		4 seasons	Parvin molaei zadeh	2014	2016
Golestan	6	209	1.89	7.55–8.46	4 seasons	Ali shahriari	2009	2011
Urmia	3	384	0.6	7.5–8.3		Hasan nan bakhsh	2001	2005
Kashan	4	100	1.5 ± 0.7	7.7 ± 0.29	2 years	Sima rasti	1998	2011
Kerman shah	12	129	0.89 ± 1.63	7.32 ± 0.34		Afsaneh hagh morad korasti		2016
Yazd	5	240	1.3	7.6	Summer and winter	Mohammad Hossein kargar	2007	2008
Bandar abbas	5	15	1.68	7.2–7.9	ND	Zohreh kherad pisheh	2010	2012
Tabriz	44	352	0.76 ± 1.95	8.2 ± 0.29	1 year	Mohammad mosaferi	2016	2018
Golstan	8	1039	2.5 ± 1.35	7.8 ± 0.34	5 years	Yazdanbakhsh	2009–2013	2016
Golestan	8	291	1–3.5	7.2–8	5 Jun to November	Manshori	2010	2015
Kerman shah	24	99	1.10			Yunes sohrabi	2016	2017
Kashan	4	200	1.5 ± 0.7	7.7 ± 0.29	4 seasons	Sima rasti	2011	2012
Shahrekord	5	21	1.62	7.8	winter	Shirin saberian poor	2014	2015
Hamedan	4		0.84 ± 0.5	7.38 ± 0.5	Februay to May	Edris Hossein zadeh	2011	2013
Sanandaj	16	128	1.01 2.03	7.2–7.7 6.7–7.6	June to November	Pegah Bahmani	2016	2018
Shiraz	13		1.5	7.6	Spring and summer	Fatemeh ghasemi	2016	2019
Arak	6	576	1–3	7.2–8	4 seasons	Hossein sarmadian	2013–2014	2020

According to Table 2, 19 of these 33 studies only measured chlorine and pH in 14 different provinces of Iran.

**Fig. 3:**
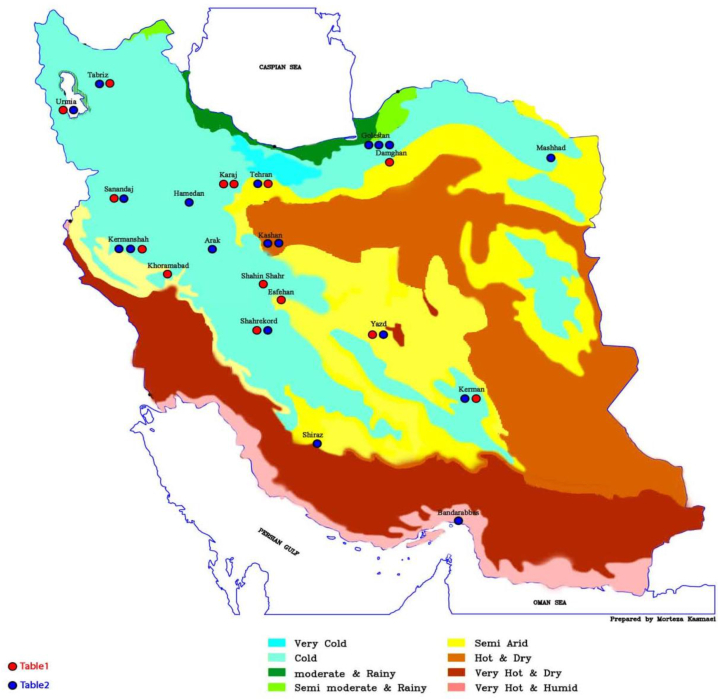
The location of pools according to Iran's climate in different cities.

All the studied pools were covered and were active throughout the year. However, the reason for seasonal sampling in some studies was due to the conditions and situation considered by the researchers of the authors. According to the existence of four types of climates in Iran, all studies were categorized in these four types of climates Fig. 3.

The free chlorine in the water of Iranian swimming pools was varies widely. The mean and deviation of the residual free chlorine in 14 studies (Table 1) and19 studies (Table 2) were equaled to 1.63 ± 0.59 mg/L, and 0.53 ± 1.56 mg/L respectively which were below the residual free chlorine standard for pool water (1–3 mg/L). However, in the studies of Sohrabi et al., Yazdanbakhsh et al. in Golestan, as well as the study of kamerei et al. in Khorramabad, and the standard deviation of the residual chlorine concentration, the residual concentration has gone higher than the permissible limit, Also, the mean chlorine concentration in 25 % is lower than the standard, however, in 66 % of the studies the remaining chlorine concentrations of swimming pools are at the permissible limit.

According to the results of the study of Rezai et al., more than 60 % of swimming pools in Shahinshahr had the chlorine values less than 1 mg/L [22]. According to the Pearson correlation coefficient, there was a significant relationship between the remaining free chlorine, pH, and turbidity. Increasing turbidity is not only the main cause of pH increase but also reduces the effect on germs due to increased hypochlorite ions and reduced chlorine. The results of three swimming pools in Shahin Shahr showed that the lowest percentage of the separated bacteria were related to the coliform, and in all pools, the samples were less than the standard but the whole fecal coliform and heterotrophic plate counting in one pool, as well as *Pseudomonas aeruginosa* in two other pools, had exceeded the standard. Based the Pearson correlation coefficient between the free chlorine and the total coliform is reversible significant relationship (p<0.05) (20).

According to the results of Beiki et al., the average *heterotroph bacteria* in Tehran pool water were 154.5 CFU/ml and in 8.6 % of cases above the standard. There was also a high correlation between *HPC* and *thermophilic Coliform* ($R^{2}>0.91$). The average free chlorine residual pools in Tehran were 1.8 mg/L and 82.8 % of the pools were in the standard range [23]. Also, in the study of the Dehghani et al., on 27 pools in Tehran, 62.5 % of microbial contamination (*HPC* positive test) was observed when the free chlorine was zero and was observed and was observed and was found zero, and 29.2 % of microbial contamination (negative HPC test) in the remaining free chlorine between zero and 1 mg/L or above 3 mg/L and only 8.3 % microbial pollution was when the remaining free chlorine was in the standard limit [24]. This study only stated the presence or absence of heterotrophs in the pool water, without mentioning the number, so it was in Table 2.

In the study of Moeinian et al., on the men and women swimming pools in Damghan, the average remaining chlorine in the pool of men and women was 1.47 and 2.47 mg/L respectively. Also, the average heterotrophs and coliforms in both men and women pools were more than permissible, which was reported in the children pool more than in the men pool. In this study pearson test indicated the reverse correlation between the remaining chlorine with *heterotrophs*, with the increase in the residual chlorine righteous, the number of index bacteria decreased [17].

The results of the microbial quality of water swimming pools in Urmia in the study of Zazouli et al. Show that in some samples *heterotrophic* and coliform *bacteria* were higher than the standard. Also, pearson correlation test was showed the reversible relationship between the residual chlorine and the *heterotrophic bacteria, Staphylococcus aureus, Pseudomonas aeruginosa, Streptococcus,* and *total* coliforms [25]. The comparison of two studies of swimming pools in Urmia in the years 84 and 93 indicates the improvement and standardization of the residual free chlorine status, *heterotrophs*, and pH [15,25]. In the study of the residual free chlorine rate in 76.143 % of the study, there was an inappropriate relationship between the HPC and free chlorine (p<0.026) [26].

In the study of Yazdan Bakhsh et al. on 8 pools equipped with a Jacuzzi in Golestan province, the average chlorine value in the total pools was 2.5 ± 1.35 mg/L, and in the total jacuzzi 1.3 ± 1.5 mg/L. The residue in the swimming pools was higher than in the Jacuzzi. In this study, there is a significant correlation between the amount of free chlorine and all the coliform, both in swimming pools and in the jacuzzi, so that the higher the amount of chlorine, the lower the number of these microorganisms [27]. The mean amount of free chlorine in the study of Molazade et al. in Kerman was 1.6 mg/L and is 45 % above the standard. In addition to the remaining free chlorine, other factors are also effective in achieving optimal disinfection efficiency.

The results of this study also showed that jacuzzi water was more contaminated than other pool parts due to its lower volume than other parts of the pool [28]. In a Mosaferi et al. on swimming pools in Tabriz, the remaining chlorine in 13 % of the main pools, 81.2 % of jacuzzi pools, 71.4 % of chiller pools, and 12.5 % of children's pools (1–3 mg/L) (10). Also, the results of Firouzi et al. study in Tabriz's pools water was showed that HPC in the disinfectant pools with chlorine was $200-68\times 10^{4}Cfu/ml$ and disinfectant pools with chlorine ozone (CHDP- O3) HPC number of 80 % was higher than the national standard of Iran and WHO instructions [10]. In the study by Bahmani et al., on the of sanandaj pools; the mean of free chlorine residue was 1.5 mg/L however *heterotroph bacteria*, *Pseudomonas aeruginosa*, and *Streptococcus* fecalis were lower than the standard in all samples, and statistical analysis was showed significant relationship between the remaining chlorine and the fecal and total coliform [29].

In the study of Jafari Nia et al., the free chlorine in 88.9 % pools in Karaj city was standard and desirable range, and the number of *heterotrophic bacteria* in 99 % of the samples was negatively. Also, a significant relationship was observed by the Pearson correlation test between *heterotroph bacteria* with water turbidity and area to a swimmer, pool area, and pool capacity, with increased opacity and reduced area to a swimmer [30].

The pH standard in the swimming pool water is between 7.2 and 8, which is 25 % of the studies in Iran are less than or higher than the standard. minimum and maximum pH reported in Iranian pools was 6.1 and 9.5 [11]. In the study by Farzad Kia et al., the HPC in all samples of pool water and hot water resources in North Khorasan province, was more than the permissible value (200 CFU/ml) while mean and std of HPC in the inlet water source was 137 ± 30 and less than the standard limit [31]. Turbidity as an important parameter affects the disinfectant and its increase causes the effect of disinfectants to decrease, in some studies was greater than the standard [7].

In all the published articles, the water entering the swimming pools is from a well or city piped and purified water, which after entering the swimming pool is heated in the swimming pool installation unit and then disinfected with chlorine and enters the swimming pool. And in all chlorine control studies, it is carried out and monitored continuously with the supervision and emphasis of health organizations. Although UV lamps and ozone are also used in some pools, chlorination and its monitoring are among the health requirements of pool water.

## 4 Discussion

In this systematic review, the relationship between the residual free chlorine concentration and heterotrophs in the swimming pools water of Iran was evaluated. Various factors can affect the residual free chlorine in the swimming pool water including water temperature, number of swimmers, pH, coliforms, and heterotrophs. Meanwhile, the only variable that can show the reliability of the water microbial quality is the residual free chlorine concentration [[32], [33], [34]]. But the monitoring studies of swimming pools indicate a significant correlation between the free chlorine and heterotrophs, which decreases the free chlorine concentration. According to various studies, heterotrophs in pool water consume organic substances, especially chlorine compounds, for growth and survival. As a result, there is a significant relationship between free chlorine levels and heterotrophs. This decrease in the concentration of free chlorine weakens the disinfecting power of the pool water. This can lead to increase the waterborne illnesses and infections for swimmers. By maintaining adequate levels of free chlorine and implementing proper pool maintenance practices, the growth of heterotrophs can be controlled and the safety of the pool water can be ensured [2,33,35,36]. However, in several different studies, the microbial status of the pool water has been reported due to the low free chlorine concentration. There can be several factors contributing to low free chlorine levels in pool water. One common reason is the lack of proper maintenance and regular monitoring of chlorine levels. In addition, excessive use of the pool can drain the free chlorine in the water more quickly and reduce its effectiveness, when a large number of swimmers enter the pool with organic substances such as sweat, urine and body oils. These organic compounds can react with the free chlorine, and leading to its depletion.

Another effective factor in the low level of free chlorine in the pool water is insufficient filtration and circulation systems. Inadequate filtration leads to ineffective removal of particles and pollutants from water and increases the demand for chlorine for disinfection. In addition, poor water circulation leads to the creation of local areas with low chlorine concentration and the growth of bacteria in those areas [37,38]. However most iranian studies, the remaining chlorine range is in the standard study, but the study in Athens showed that only 27 % of the samples harvested from the pool water are free of chlorine. Some pools may not be chlorinated enough, potentially leading to an increased microbial load in the water [39]. In a study from Florida, 486 pools found that 22 % of the remaining free chlorine was less than 1 mg/L, which is consistently consistent with the current systematic study results that 25 % expressed [33]. Therefore, regular and continuous pool chlorination is necessary to prevent the proliferation of harmful microorganisms and to ensure the swimmers health [40]. In addition to the residual chlorine concentration, adjusting the pH of the pool water is also necessary to achieve optimal disinfection. In 25 % of these studies, the pH levels in the pools do not match the standard range. This non-compliance significantly reduces the effectiveness of chlorine in improving the microbial quality of water, and if the pH was not balanced properly, chlorine becomes less effective at killing harmful bacteria in pool water [2,5,6,8,10,41].

High pH in pool water can lead to several issues, one of the major concerns is the flooding of pool walls, water supply pipes, and sand filters. The high pH causes scaling and precipitation of minerals, and finally it causes blockage and reduction of water flow. This can increase maintenance and repair costs. In addition, high pH require an increased amount of chlorine, leading to increasing the chlorine consumption and the operational and maintaining costs. In other hand, excessive chlorine usage can result to eyes and skin irritation of swimmers [25]. Also increase in pH lead to producing the hypochlorous acid, and reducing the chlorine disinfection, also low pH can also cause skin irritation, dryness, and itching of swimmers. Therefore, regular monitoring of pH levels is essential [28]. One of the variables affecting the disinfection of swimming pool water is turbidity, which has been measured in a limited number of input studies, and according to the Pearson correlation coefficient, it has a significant relationship with the reduction of residual chlorine and the increase of water pH (<0.05), in this regard, water clarity along with residual free chlorine concentration is a suitable measure to ensure the bacteriological quality of water. Continuous cleaning of the floor and walls of pool the turbidity reduction and maintains the remaining free chlorine concentration, thus increasing the water bacteriological quality [22,33].

In some studies, the water quality of Jacuzzi was investigated, and it was found that Jacuzzi water is more polluted than other parts of the pool due to its smaller volume [12,28]. Also, higher temperature of water in the Jacuzzi causes the chlorine to dissipate faster. In addition, the density of swimmers in a smaller space increases the organic load in the water, which requires a higher concentration of chlorine to effectively control the existing microorganisms. Therefore, regular monitoring and appropriate control measures, such as maintaining free residual chlorine concentration, are necessary to achieve optimal disinfection and minimize the risk of waterborne diseases in Jacuzzi [26]. On the other hand, the temperature of more than 29^oc^ pool water causes weakness in the swimmers, while it causes the growth of green algae and microorganisms and damages the disinfection operation. Chlorine or other disinfectants used in swimming pools are less effective at higher temperatures, reducing their ability to kill harmful bacteria and viruses. This is dangerous for the health of swimmers [11,42]. The temperature of thejacuzzi in Ghaneian's study in Yazd was 38^oc^, and in the study of Mosaferi in Tabirz, the temperature was 41.1^oc^, which is higher than the standard, of course, because of the interest and request of Iranian swimmers and their request to increase the temperature.

Comparison between the seasons in the study of Firouzi et al., on the Tabriz's water pools was shoed the HPC in the autumn were less than summer and winter. Lower HPC during autumn is positively correlated with lower turbidity and better water quality compared to summer and winter. However, lower temperatures during the fall prevent the growth of microorganisms and limit their population in the pool water [10]. In order to ensure the effective disinfection of pool water and to eliminate microorganisms, it is very important to maintain optimal water conditions, and physicochemical parameters play an important role in the effectiveness of the disinfection process. On the other hand, the failure of the chlorination system has been reported in some studies, which has disturbed the concentration of chlorine in the water, which has proven the need for a maintenance system. In addition, the presence of nitro ammonium and organic compounds in water and its combination with chlorine reduces the concentration of chlorine, therefore, regular monitoring and removal of any unwanted pollutants in the pool water that can interfere with the disinfection process is very important [43].

It should be mentioned that excessive use, reduction of pool volume, high temperature of jacuzzi, increase of turbidity and continuous decrease or increase of pH are considered as critical conditions due to the adverse health consequences it has on swimmers and if observed during health monitoring, lead to the closing of the swimming pool.

Due to the fact that pools are divided into three shallow, semi-deep and deep zones, measurements of physical and chemical parameters are usually recorded in most studies. In most of the studies, measurements of chlorine, turbidity and coliform have been performed at different depths of the pool, but a limited number of studies have been performed to measure heterotrophs at different depths of the pool. According to the results of published articles, the concentration of combined chlorine, turbidity, heterotrophs and coliform in deep areas is higher than in shallow areas and more than in children's pools. Also, these values are higher in the men's pool than the women's pool. The possible causes are due to the accumulation of water in the deep area and the short time of swimming in the children's pool. In addition, the incoming water has more disinfection properties and there is fresher water in the shallow part.

One of the important things in the input studies is the reduction of residual chlorine levels and the growth of algae, which aggravates the turbidity of the water, and leads to the creation of slimy surfaces on the walls of the pool, steps and other structures. Therefore, the visual appeal of the pool is reduced and it also brings the risk of slipping.

In entry studies, various factors such as low water chlorine level, high pH and high alkalinity of water, high calcium hardness, high water temperature and low circulation or incomplete filtration of water have been reported as the causes of increased turbidity. In the study of Shahin Shahr pool, the correlation between turbidity and heterotrophs was 0.94 and the correlation between residual free chlorine and turbidity was reported as 0.594. Also, in the study of Kerman pools by Jafari Mansourian et al., the correlation between residual free chlorine and turbidity was reported. It was 0.594. The turbidity and the sum of heterotrophic microorganisms and Pseudomonas were reported to be 0.345 and the correlation between residual free chlorine and turbidity was 0.094. Therefore, according to most of the studies conducted, in general, an inverse relationship between residual chlorine levels and the studied organisms was observed. This shows that the population of heterotrophic microorganisms and Pseudomonas tend to decrease with the increase of residual chlorine concentration in pool water. The inverse relationship also indicates that residual chlorine acts as a disinfectant and helps control the growth of these microorganisms in the pool water.

In this review systematic study; all the studied pools were public and entry to them requires paying a fee, and the cost of swimming in Iran's public pools was almost the same. Only the management of the studied swimming pools, whether private or public, that according to the health monitoring by inspectors, there is no difference in checking the quality of the water of the pools in terms of private or public. And only in the study of Jafari nia et al., he said that there were more bacteria in the public pool than in the private pool. On the other hand, there are private pools and pools in some areas of several Iranian cities, but these pools have not been studied in published articles.

One of the most important issues affecting the quality of swimming pool water is the number of swimmers, which can become a health problem if the number of swimmers is not proportional to the capacity of the pool. Unfortunately, this factor has not been investigated in the published articles. However, controls and monitoring during continuous inspections in pools by Iran's environmental health experts have minimized this problem. In addition, in the studied swimming pools in the published articles, the pool operator tries to control the hygiene principles as much as possible, knowing about the continuous sampling by the health inspectors.

Unfortunately, in the input studies, the operating periods of these pools and its effect on the physicochemical variables of water have not been discussed, which is one of the limitations of the study, which is considered as a limitation of this study. Therefore, it is necessary to investigate the health of swimming pools, to explain the effects of repair and maintenance programs on water quality variables. This is important in the final report of this study sent to the health authorities.

One of the most important limitations of the study is the lack of attention to the type of chlorine used in swimming pools and its consequences, which can be influenced by the type of chlorine, the amount of turbidity can be different or the amount of removal of microbial contamination can be different. In other hand, the longer the filter cycle, the less chlorine you will need. Similarly, the more chlorine you use, the shorter the filtration cycle required.

In Karami et al.'s study in Kermanshah, sand filters were used, and the results showed that *heterotrophic bacteria* were desirable in 100 % of women's swimming pools. In a study conducted by Zazouli et al. in Urmia, the usual type of treatment was screening and diatomaceous filter and disinfection with chlorine. The results indicated that 66.96 % of the samples were desirable in terms of *heterotrophic bacteria*.

In the study of Firozi et al. in Tabriz, apart from chlorine disinfection, they used chlorine and ozone disinfection, and the results of *heterotrophic bacteria* with chlorine disinfection showed that all samples were higher than the national and WHO standard, and chlorine and ozone disinfection in 83.3 The percentage of samples was higher than the standard.

It is suggested; Based on the results and conclusions of published articles, it is necessary to implement the operation and maintenance system for pools and operational planning for the pool. Based on this, it is necessary to train the operator of the facility in the field of common chlorination and shock, as well as explain the importance of environmental and operational conditions on water quality. Also, in order to improve the performance of the pool, maintenance and filtration control operations must be done carefully. In addition to regular disinfection, proper operations and maintenance activities should also be undertaken like frequent filter backwashes, sweeping of pool, removing organic matter (leaves, debris) and refill.

## 5 Conclusion

The results of statistical tests in a number of articles included in this study have shown that the reverse correlation between the remaining chlorine with heterotrophs, and with the increase in the chlorine, the number of index bacteria decreased. Also, the results of the present study showed that in pools where the level of residual free chlorine is suitable, heterotrophs are controlled, and vice versa. Therefore, the expansion of control measures based on the variables mentioned in the study to maintain the residual chlorine level can be effective in controlling heterotrophs in recreational pools. In addition, results of the present systematic study showed that in most studies, the residual free chlorine was high. So to operate swimming pool more efficiently, regular monitoring to comply with chlorine levels, and maintaining the pH range to improve the efficiency of free chlorine and heterotrophy in water Swimming pools are required.

## Funding declaration

No funding has been received.

## Data availability

Data are available from the corresponding author upon request.

## CRediT authorship contribution statement

**Yasaman Oshidari:** Investigation. **Vida Amoohadi:** Investigation. **Hadi Niknejad:** Investigation. **Reza Zeraatkar:** Writing – review & editing. **Mohsen Hesami Arani:** Writing – original draft, Methodology.

## Declaration of competing interest

The authors declare that they have no known competing financial interests or personal relationships that could have appeared to influence the work reported in this paper.
